# Divalent Cations (Ca^2+^, Mg^2+^, Mn^2+^, Fe^2+^, Ni^2+^, and Zn^2+^) Enhance Growth of *Xanthomonas citri* and *X*. *campestris* by Reducing Generation Time

**DOI:** 10.1021/acsomega.5c02786

**Published:** 2025-08-06

**Authors:** Davi Gabriel Salustiano Merighi, Cauê Augusto Boneto Gonçalves, Anielle Salviano de Almeida Ferrari, Maxuel de Oliveira Andrade, Cristiane Rodrigues Guzzo

**Affiliations:** † Department of Microbiology, Institute of Biomedical Sciences, University of São Paulo, São Paulo CEP 05508-000, Brazil; ‡ Brazilian Biorenewables National Laboratory (LNBR), Brazilian Center for Research in Energy and Materials (CNPEM), Campinas 13083-970, Brazil

## Abstract

Bacterial ion homeostasis is critical for adaptation and survival. In this study, we show that supplementing 2xTY medium with divalent cations (Ca^2+^, Mg^2+^, Mn^2+^, Fe^2+^, Ni^2+^, and Zn^2+^) from 50 to 1,000 μM significantly enhances the growth of *Xanthomonas* species by reducing generation time, while monovalent ions (Na^+^, K^+^, Li^+^, and Cl^–^) had no significant effect. Among them, Ca^2+^ effectively boosted *X*. *citri* fitness, increasing the specific growth rate by up to 223%, shortening the cell cycle, and enhancing adhesion to abiotic surfaces up to 8-fold. Calcium also induced a more uniform and smaller cell morphology, suggesting improved cell division and metabolic efficiency. Colony forming unit counts confirmed a higher viability, excluding dead cell accumulation. These effects were conserved in other Xanthomonadaceae members (*X*. *campestris* and *S*. *maltophilia*) but not in nonrelated species. Based on these findings, we propose an optimized growth medium named 2xTY-Ca (16 g/L tryptone, 10 g/L yeast extract, 5 g/L NaCl, and 1 mM CaCl_2_), which significantly improves *Xanthomonas* spp. growth kinetics *in vitro*, enhancing *X*. *citri* and *X*. *campestris* cultivation for laboratory and industrial applications. Though the precise molecular mechanism behind these phenotypes has yet to be determined, we highlight *X*. *citri* as a promising organism to understand the role of divalent cations in bacterial physiology.

## Graphical abstract

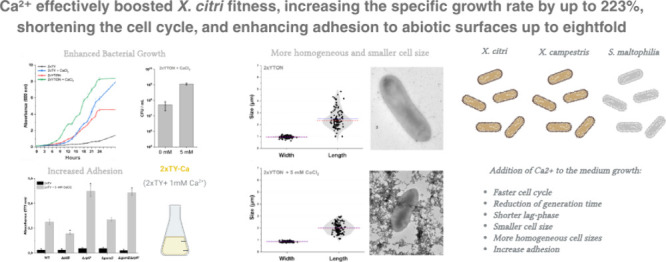

## Introduction

Every form of life has evolved to acquire ions from the environment and use them in fundamental physiological processes. These macronutrients, micronutrients, and trace metals are essential to the correct bacteria fitness, replication, and response to environmental conditions. In biology and biochemistry, monovalent ions are associated with homeostasis, electrical activity, membrane potential regulation, osmotic adaptation, ion transport, and protein folding,^1,2^ while divalent ions play important roles in the structural, catalytic, and regulatory activity of proteins, cellular signaling, sporulation, and membrane stabilization.^3–5^

Energy generated by the electron transfer chain during respiration is used to pump H^+^ across the membrane, resulting in a potential charge difference in the cytoplasmic cell membrane named proton-motive force or electrochemical potential.^6^ The inward-directed proton-motive force is related to ATP synthesis, whereas proton extrusion is responsible for membrane potential and intracellular pH management.^7^ Flagellar motility, for example, is dependent on the electrochemical potential generated by the influx of H^+^ through MotAB stator proteins acting as energy sources for flagella rotation.^8^ In addition to H^+^, some bacteria can use Na^+^ as a coupling ion to generate the sodium-motive force.^9^ A variety of membrane carrier proteins play pivotal roles in regulating cytoplasmic ion concentrations through mechanisms such as uniporters, symporters, and antiporters. One prominent example includes K^+^/Na^+^ exchangers, which are crucial for maintaining cellular ion balance by exchanging potassium and sodium ions across cell membranes.^10^ In *Streptococcus faecalis*, it was observed that during the logarithmic growth stage, the bacterium extrudes Na^+^ ions while accumulating K^+^ ions in the cytoplasm. However, the intracellular concentrations of Na^+^ and K^+^ normalize to similar levels during the stationary phase.^11^ Moreover, K^+^/Na^+^ exchange is also an important strategy employed by halophiles to reduce the intracellular level of Na^+^ and accumulate K^+^ to balance osmotic pressure.^12^

Intracellular concentrations of divalent cations such as Ca^2+^, Mg^2+^, and Mn^2+^ are tightly regulated in bacteria and play essential roles in cellular physiology and adaptation. Cytoplasmic Ca^2+^ is maintained between 100 and 300 nM,^13–15^ typically lower than extracellular levels, while it can accumulate in the periplasm,^16^ sometimes exceeding external concentrations. Acting as an intracellular messenger, Ca^2+^ mediates responses to environmental stress by modulating motility,^17^ virulence,^18^ and heavy metal tolerance.^19^ Mg^2+^, one of the most abundant intracellular ions after K^+^, is critical for homeostasis and serves as a cofactor in various processes, including enzyme function and the stabilization of nucleic acids, ribosomes, and membranes.^20,21^ In contrast, Mn^2+^ is required in trace amounts in many bacteria, especially under stress,^22^ but can reach cytoplasmic concentrations up to 35 mM in species with manganese-centric metabolism, such as *Lactiplantibacillus plantarum*.^23,24^

Here, we report that *Xanthomonas citri* pv. *citri* 306 growth is significantly enhanced by supplementing the medium with divalent ions such as Ca^2+^, Mg^2+^, Mn^2+^, Fe^2+^, Ni^2+^, and Zn^2+^, reducing the generation time and lag phase and enhancing growth. In contrast, we did not detect any effect in the presence of monovalent ions (Na^+^, K^+^, Li^+^ and Cl^–^) in the bacterial growth. We found that divalent ions also influence bacterial morphology and confer halotolerance to *Xanthomonas* species. Interestingly, this feature seems to be restricted to the Xanthomonadaceae family since *Xanthomonas campestris* pv. *campestris* ATCC 33913 and *Stenotrophomonas maltophilia* K279a could also benefit from calcium supplementation but not bacteria belonging to other classes (Bacilli, Gammaproteobacteria, Alphaproteobacteria, and Spirochaetia). Moreover, we found that while Na^+^ itself is not capable of enhancing growth, it is necessary, jointly with calcium, for stationary phase stabilization, preventing an early decline phase. We, therefore, present a novel growth medium (named in this work as 2xTY-Ca) for *Xanthomonas* species that increases the final cell density up to 5-fold compared to the standard 2xTY medium.

## Materials and Methods

### Bacterial Growth Conditions

*Xanthomonas citri* pv. *citri* 306, *Xanthomonas campestris* pv. *campestris* ATCC 33913, *Acinetobacter baumannii* ATCC 19606, *Staphylococcus aureus* ATCC 25923, *Escherichia coli* K-12, and *Stenotrophomonas maltophilia* K279a were grown in 2xTY (16 g/L tryptone, 10 g/L yeast extract, 10 g/L NaCl, pH 7.5), 2xYTON (16 g/L tryptone, 10 g/L yeast extract, pH 7.5), and a new medium named in this work as 2xTY-Ca (16 g/L tryptone, 10 g/L yeast extract, 5 g/L NaCl, 1 mM CaCl_2_, pH 7.5) at 28 °C under agitation. *Caulobacter crescentus* NA1000 was grown in PYE medium (2 g/L peptone, 1 g/L yeast extract, 0.5 mM CaCl_2_, 1 mM MgCl_2_, pH 7.5). Solid medium was prepared by the addition of 15 g/L Difco bacterial agar. *Leptospira biflexa* serovar Patoc strain Patoc1 (Ames) was cultured in commercial *Leptospira* Medium Base Ellinghausen–McCullough–​Johnson–Harris (EMJH - BD Difco, Sparks, MD, USA), supplemented with *Leptospira* Enrichment EMJH (BD Difco, Sparks, MD, USA), at 28 °C without agitation for 5 days, until reaching the mid-exponential phase. The initial culture medium pH was corrected using HCl or NaOH, monitored with a pH meter (BEL Engineering). Experiments in which preinoculum was described as “overnight cultures” indicates an incubation period of 12–16 h prior to experimentation.

### Growth Curves in Microplate

Growth curves were performed in 96-well flat-bottom microplates and read in a Synergy H1 Hybrid Multi-Mode microplate reader (BioTek, Winooski, VT, USA). Plates were prepared according to the following scheme: Lines A and H and columns 1 and 12 were filled with 150 μL of autoclaved Milli-Q water to maintain the humidity. The experiment was prepared by pipetting 90 μL of culture medium and 10 μL of mid-log growth phase bacteria adjusted to an OD_600 nm_ of 0.6, in triplicate or quintuplicate. Three to five wells were filled only with culture medium as a blank control. The culture in the plates was incubated at 28 or 37 °C under orbital agitation, and the OD_600 nm_ was measured each hour. The *L*. *biflexa* growth curve was performed by incubating in 5 mL of EMJH medium as described above in glass tubes and daily counted in a Neubauer chamber using a dark-field optical microscope Nikon Eclipse E200. Data were processed in Excel, and graphs were generated using Origin (version 2022b).

### Calculation of Generation Time (*g*)

Growth curve data were plotted in a logarithmic log_10_ format. The lag phase was defined as the period from the initial measurement (0 h) to the last time point with no increase in absorbance immediately before the onset of exponential growth. The exponential growth phase was determined by identifying the first time point where absorbance increased following the lag phase and the last time point before the stationary phase when it reached the maximum absorbance and the curve entered a plateau. To calculate the generation time, five consecutive data points from the mid-exponential phase were selected, excluding the first and last two or three points, depending on the bacterial species. The log_10_ of these OD_600 nm_ values was calculated and used to construct a semilogarithmic plot (time on the *x*-axis and log_10_ OD_600 nm_ on the *y*-axis). A linear regression was performed, and only data sets with *R* ^2^ > 0.95 were considered. The generation time (*g*) was calculated using the following equation:

$$y=\frac{\log2}{g}$$

yg=log⁡2

$$g=\frac{\log2}{y}$$

Since log_10_ 2 ≈ 0.301, this simplifies to

$$g=\frac{0.301}{y}$$

where *g* is the generation time and *y* is the slope of the exponential phase in the semilogarithmic plot. Data were processed in Excel, and graphs were generated using Origin (version 2022b).

### Calculation of Specific Growth Rate (*k*)

The specific growth rate (*k*) was calculated based on the generation time (*g*), using the following equation:

$$k=\frac{0.301}{g}$$

Therefore, the bacterial growth enhancement was quantified according to the percent increase of *k*, obtained using the following equation:

$$\mathrm{percent increase of}\;k\;\left(\%\right)=\frac{k_{1}-k_{0}}{k_{0}}\times 100$$

$$\mathrm{percent increase of}\;k\;\left(\%\right)=\frac{\frac{0.301}{g_{1}}-\frac{0.301}{g_{0}}}{\frac{0.301}{g_{0}}}\times 100$$

$$\mathrm{percent increase of}\;k\;\left(\%\right)=\frac{g_{0}-g_{1}}{g_{1}}\times 100$$

where *k*_0_ is the specific growth rate of the bacteria in the control medium (without ion addition) and *k*_1_ is the specific growth rate of the bacteria in the medium supplemented with the respective ions. Values of *k* were calculated using *g* rounded to the first decimal place.

### Colony Forming Unit (CFU) Counting

An overnight culture of *X*. *citri* WT grown to mid-log phase at 28 °C under agitation was used to inoculate fresh 2xYTON medium, with or without supplementation of monovalent and divalent cations. The bacterial culture was adjusted to an initial OD_600 nm_ of 0.1 in a final volume of 5 mL. After incubation for 12 h under the same conditions, bacterial suspensions were serially diluted in fresh 2xYTON medium, plated on 2xYTON agar, and incubated for 48 h for colony formation unit (CFU) quantification. The bacterial concentration (CFU/mL) was calculated using the following equation:

$$\mathrm{CFU}/\mathrm{mL}=\frac{\mathrm{number of colonies}\times \mathrm{inverse dilution factor}}{\mathrm{plated volume}\;\left(\mathrm{mL}\right)}$$

The experiment was performed in triplicate. Data analysis was conducted using Excel, and graphs were generated in Origin (version 2022b).

### Transmission Electron Microscopy (TEM)

An overnight culture of *X*. *citri* WT in mid-log phase grown at 28 °C under agitation was used to inoculate 5 mL of 2xYTON medium with or without supplementation by monovalent and divalent cations, adjusting the bacterial OD_600 nm_ to 0.1, and incubated for 12 h under the same conditions. The bacteria were pelleted by centrifugation at 1,200*g* for 10 min and resuspended in 100 μL of fresh prewarmed 2xYTON medium at 28 °C. A total of 5 μL of each sample was pipetted over glow-discharged 400-mesh copper grids coated with carbon film (Electron Microscopy Sciences), incubated for 1 min at room temperature, and dried with filter paper. After that, 3 μL of 2% uranyl acetate solution was pipetted on the grid, incubated again for 1 min, and dried with filter paper. The grids were analyzed using a JEOL JEM 2100 transmission electron microscope (200 kV) at the Institute of Chemistry, University of São Paulo (IQ-USP) or a Tecnai FEI G20 (200 kV) transmission electron microscope at the Institute of Biomedical Sciences, University of São Paulo (ICB-USP). Damaged cells were excluded, and dimensions of 100 bacteria were measured using ImageJ (v1.54p) software,^25^ according to the scale bar in the micrographs. The data were processed in Excel, and graphs were generated by using Origin (version 2022b).

### Adhesion Assay

Bacterial adhesion assays were performed in 96-well flat-bottom microplates following the scheme described in the Growth Curves in Microplate section. A total of 140 μL of the 2xYTON medium with or without supplementation by monovalent and divalent cations was pipetted into the microplates, followed by the addition of 10 μL of overnight cultures adjusted to an OD_600 nm_ of 0.6. *X*. *citri* was incubated at 28 °C for 5 days, while *E*. *coli*, *A*. *baumannii*, and *S*. *aureus* were incubated at 37 °C for 48 h, all under static conditions. Completing this step, the medium was removed, and each well was washed three times with 200 μL of PBS (8 g/L NaCl, 0.2 g/L KCl, 1.44 g/L Na_2_HPO_4_, 0.245 g/L KH_2_PO_4_, pH 7.4). The plates were then dried at 28 °C for 15 min. Bacterial biofilms were stained by adding 175 μL of 0.1% crystal violet to each well, followed by a 15 min incubation at room temperature. The staining solution was removed, and the wells were washed three times with 250 μL of PBS. The retained crystal violet was solubilized in 200 μL of 95% ethanol. Wells containing only medium served as blanks. Absorbance was measured at 575 nm using an Epoch microplate reader (BioTek). Data were processed in Excel. Statistical significance was determined using Student’s *t*-test, and graphs were generated using Origin (version 2022b).

### Knockout Constructions

The *pilB*, *gumD*, and *rpfF* from *Xanthomonas citri* pv. *citri* 306 (previously named *Xanthomonas axonopodis* pv. *citri* 306) are codified by locus_tag XAC3239, XAC2583, and XAC1879, respectively, in the KEGG database. Δ*pilB*_XAC3239_ ^26^ and Δ*gumD*_XAC2583_ ^27^ mutants used in this work were kindly provided by Professor Chuck Shaker Farah from the Chemistry Institute from the University of São Paulo, Brazil. Δ*rpfF*_XAC1879_ and the Δ*rpfF*Δ*gumD* double mutant were created by homologous recombination using the primers shown in Table 1. For the construction of Δ*rpfF*_XAC1879_, approximately 1 kb upstream (F1 fragment) and downstream (F2 fragment) of the target gene preserving 30–100 bp of the *rpfF* gene were amplified using Δ*rpfF*_Forw-F1 and Δ*rpfF*_Rev-F1 for the first fragment and Δ*rpfF*_Forw-F2 and Δ*rpfF*_Rev-F2 for the second fragment. These fragments were digested using *Nco*I and ligated using T4 DNA ligase (Biolabs). The 2K fragment containing a deleted version of the gene was amplified using the primers Δ*rpfF*_Forw-F1 and Δ*rpfF*_Rev-F2, digested with *Hind*III and *Bgl*II, and cloned into the suicide vector pNPTS138 (GenBank: MK533795), which had been previously digested with the same enzymes. The product of ligation was transformed into the chemocompetent *E*. *coli* DH5α cell strain, and the positive clones were confirmed by DNA sequencing. This clone was used to transform *X*. *citri* cells, as described below. *X*. *citri* grown to an OD_600 nm_ of 0.5 was washed three times with Milli-Q water by centrifugation and transformed by electroporation (0.2 cm cuvette, 2 kV, 25 μF, 200 Ω) with 200 ng of plasmid DNA, allowing it to recover for 4 h in 2xTY before plating in 2xTY with 100 μg/mL Kanamycin (Kan). After 72 h of incubation at 28 °C, colonies were picked and tested in plates with 100 μg/mL Kan, 5% sucrose, or supplemented with both Kan + sucrose. After 24 h of incubation at 28 °C, colonies that could grow in Kan but not in sucrose or Kan + sucrose were selected, transferred to 2xTY broth without antibiotic for plasmid excision, and incubated for 24 h at 28 °C under agitation. The culture was plated in 2xTY supplemented with 5% sucrose and 100 μg/mL ampicillin. Colonies that formed after 24 h of incubation were picked and tested on 2xTY supplemented with Kan, sucrose, or both. Colonies grown only in sucrose, not Kan or Kan + sucrose, were subjected to PCR to evaluate the conversion of mutants (±2 kb PCR product) or restoration to wild type (±3 kb PCR product). Positive knockouts were confirmed by Sanger sequencing of the PCR products. For the creation of the double mutant, Δ*rpfF*Δ*gumD*, the mutant *rpfF* region (a fragment of 2 kb containing the knockout gene) was amplified using the primers Δ*rpfF*_Forw and Δ*rpfF*_Rev from the *X*. *citri* Δ*rpfF* mutant. The fragment was cloned into a pNPTS138 suicide vector and transformed by electroporation in a Δ*gumD* mutant. Knockout experiments were performed as described above.

**Table 1:**
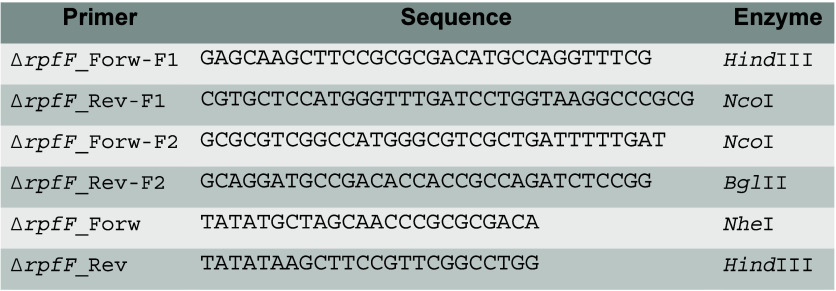
Primers Used for *X*. *citri* Knockout Constructions

### Statistical Analysis

Each assay was performed with 3–5 experimental replicates. For statistical analysis, data were processed in Excel, and statistical significance was determined using Student’s *t*-test. Graphs were generated using Origin (version 2022b).

## Results

### Divalent Cations Enhance *X*. *citri* and *X*. *campestris* Growth

During our routine experiments, we observed an improvement in the *X*. *citri* growth when incubated in a rich medium supplemented with calcium, raising our curiosity to investigate this phenotype. To evaluate the importance of different ions for this bacterium, growth curves were prepared in 2xYTON medium, which had the same composition as 2xTY but lacking NaCl supplementation, to study the ions independently of the addition of sodium, in the presence and absence of additional monovalent and divalent cations. The experiments were performed in these media once it was the most favorable conditions for growth, in comparison to minimal media where *X*. *citri* exhibited some difficulties in replication and an excess of calcium precipitates with phosphate.

Growth curves were generated for all tested ions across concentrations ranging from 10 nM to 10 mM, confirming that the addition of monovalent cations did not enhance bacterial growth at any concentration (Figure 1A–C). In contrast, all divalent cations promoted bacterial growth when supplemented at micromolar (μM) to millimolar (mM) concentrations. To determine the minimal effective concentration, we narrowed the range for divalent cations to 50–1,000 μM. Notably, Fe^2+^, Ni^2+^, Zn^2+^, and Co^2+^ were toxic at concentrations exceeding 1 mM, likely due to oxidative stress (data not shown).

**Figure 1:**
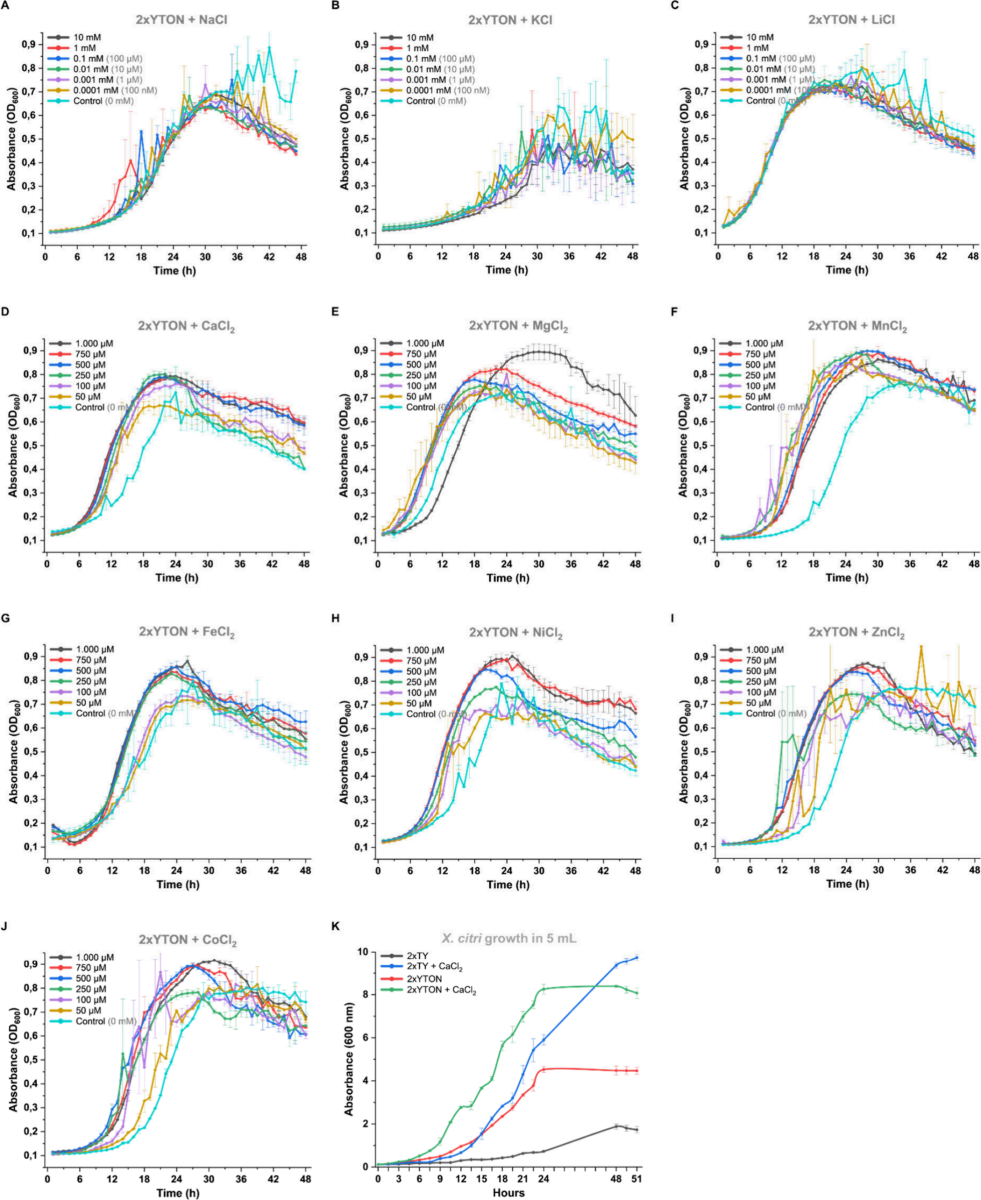
*X*. *citri* growth curves in the presence of monovalent and divalent cations. Growth curves of *X*. *citri* pv. *citri* 306 WT in 2xYTON medium supplemented with different concentrations of cations, ranging from 100 nM to 10 mM for NaCl (A), KCl (B), and LiCl (C), and from 50 to 1,000 μM for CaCl_2_ (D), MgCl_2_ (E), MnCl_2_ (F), FeCl_2_ (G), NiCl_2_ (H), ZnCl_2_ (I), and CoCl_2_ (J). Growth curve of *X*. *citri* pv. *citri* 306 grown in 5 mL of 2xTY or 2xYTON medium with and without supplementation by 5 mM CaCl_2_ (K). Three experimental replicates were performed.

Supplementing 2xYTON medium with divalent cations (Ca^2+^, Mg^2+^, Mn^2+^, Fe^2+^, Ni^2+^, and Zn^2+^) positively influenced bacterial growth, while the addition of K^+^, Na^+^, or Li^+^ did not result in any effect. Incubation with 10 mM EGTA, a chelating agent with specific affinity for Ca^2+^ ions, completely reverted this phenotype, inhibiting bacterial growth in 2xTY and decreasing it in 2xYTON (Figure S1A,B, respectively). Since all the salts used contained chloride as the counterion and calcium chelation by EGTA abolished the observed growth enhancement, it is likely that the improved bacterial fitness under the tested conditions is attributable to the specific cation supplemented, rather than the presence of chloride. Curiously, a decrease in the absorbance values soon after reaching the highest absorbance value (stationary phase) in all conditions of the 2xYTON medium may be related to a more immediate decline phase (death phase), which was not observed in 2xTY when Ca^2+^, Mg^2+^, or Mn^2+^ was present, suggesting some important role of Na^+^ in the stabilization of bacterial growth in the stationary phase.

The three main phenotypic effects most observed when *X*. *citri* was incubated in media supplemented with divalent cations were (i) a shortened lag phase; (ii) reduced generation time, reflecting a faster replication cycle; and (iii) increased final turbidity, likely due to a higher cell density in suspension. Moreover, all tested divalent cations reduced the generation time in microplate assays, thereby increasing the bacterial specific growth rate (*k*) at the best divalent cation concentration as follows: 500–1,000 μM CaCl_2_ (72.7%), 250–750 μM MgCl_2_ (35.3%), 50–100 μM MnCl_2_ (91.3%), 500–1,000 μM FeCl_2_ (96.9%), 500–1,000 μM NiCl_2_ (75%), 500–1,000 μM ZnCl_2_ (31.3%), and 500–1,000 μM CoCl_2_ (72.7%). In 5 mL cultures supplemented with 5 mM CaCl_2_, *k* was increased by up to 223.5%.

Cell populations were quantified by determining colony forming units (CFU) per milliliter. None of the three monovalent ions tested (K^+^, Na^+^, and Li^+^) led to an increase in CFU compared to the control grown in 2xYTON without ion supplementation. In contrast, supplementation with most of the tested divalent cations (Ca^2+^, Mg^2+^, Mn^2+^, Fe^2+^, Ni^2+^, and Zn^2+^) significantly increased cell concentrations relative to the control, confirming that the higher turbidity observed in Figure 1 was due to enhanced bacterial proliferation and the presence of viable cells, rather than the accumulation of dead cells in suspension or filamentation (Figure 2). Curiously, despite a positive effect in 96-well microplate growth, supplementation by CoCl_2_ resulted in CFU values similar to those of the control, suggesting that Co^2+^ does not increase the cell density like the other divalent cations (Figure 2K). In addition to *X*. *citri*, growth enhancement by calcium appears to be a conserved feature in the *Xanthomonas* genus, as it was also shown to be an important micronutrient for *Xanthomonas campestris* pv. *campestris* ATCC 33913 (Figure S2).

**Figure 2:**
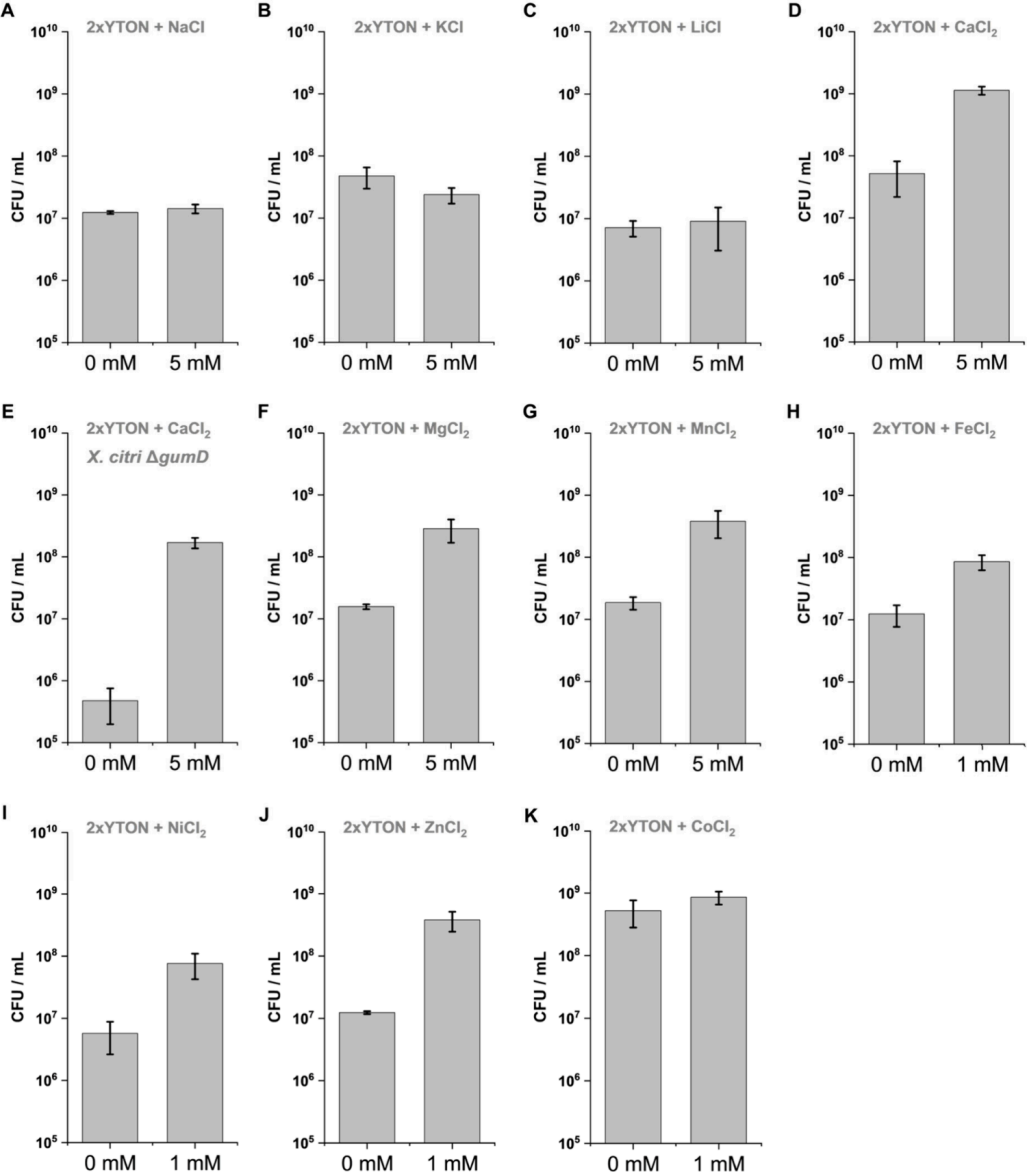
*X*. *citri* CFU counting in the presence of monovalent and divalent cations. Overnight cultures were used for inoculation in each condition and for the control group (without ions). CFU counting of *X*. *citri* pv. *citri* 306 wild type incubated in 5 mL of 2xYTON medium with or without ion supplementation. The bacteria were incubated for an additional 12 h before plating in 2xYTON agar for CFU quantification. Cations were added in concentrations of 5 mM for NaCl (A), KCl (B), LiCl (C), CaCl_2_ (D), MgCl_2_ (F), and MnCl_2_ (G), and 1 mM for FeCl_2_ (H), NiCl_2_ (I), ZnCl_2_ (J), and CoCl_2_ (K). CFU counting of *X*. *citri* pv. *citri* 306 Δ*gumD* was performed without and with 5 mM CaCl_2_ (E). Three experimental replicates were performed. Two biological replicates (*n* = 3) were performed for the CoCl_2_ condition.

### Calcium and Sodium Cooperatively Stabilize Bacterial Growth

In the aforementioned results, we have some evidence that Na^+^ can affect the development of *X*. *citri* in a mechanism different from that the observed for Ca^2+^. In agreement with the hypothesis that growth enhancement is an exclusive feature of divalent cations and not monovalent cations, the presence of 0–100 mM NaCl (0–5.4 g/L) in the medium did not significantly affect the bacterial growth curves. However, the presence of 250 or 500 mM NaCl (14.6–29.2 g/L) significantly decreased bacterial growth (Figure 3A). A continuous decrease in the absorbance values after reaching the highest bacterial density is observed in all of the curves when *X*. *citri* is grown in a medium without calcium supplementation, indicating a premature death phase (Figures 3A, black square). Despite medium supplementation with 5 mM Ca^2+^ generating considerably less noisy curves, this sudden decrease in the absorbance values was still present (Figure 3B, black, red, and blue arrows). However, when both CaCl_2_ (5 mM) and NaCl (50–250 mM) were present, this premature decline phase was not observed (Figure 3B, black square), suggesting that both ions are required for optimal bacterial growth *in vitro*. Interestingly, CaCl_2_ also enhanced *X*. *citri* halotolerance. While 250 mM NaCl alone was toxic, limiting growth to an OD_600 nm_ of approximately 0.2 (Figure 3A, yellow line), the presence of 5 mM CaCl_2_ enabled full recovery, resulting in growth curves comparable to those observed at lower NaCl concentrations and exceeding an OD_600 nm_ of 0.5 (Figure 3B, yellow line). Therefore, a simple adaptation to the 2xTY medium will be more suitable for *X*. *citri* and *X*. *campestris* growth with the following recipe: 16 g/L tryptone, 10 g/L yeast extract, 5g/L NaCl, and 1 mM CaCl_2_. For simplification, we suggest supplementing 2xTY with 1 mM CaCl_2_, named here as the 2xTY-Ca medium.

**Figure 3:**
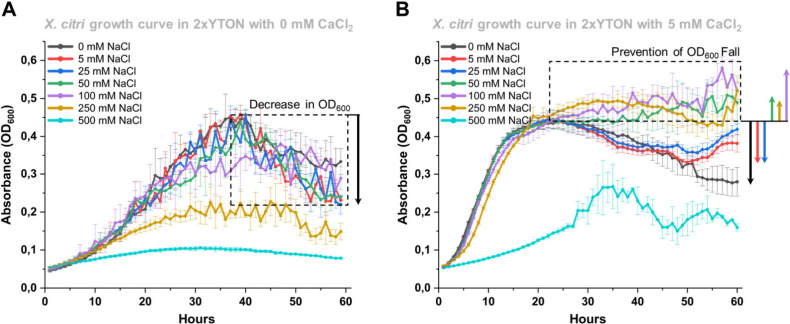
*X*. *citri* growth stabilization. (A) Influence of different concentrations of NaCl on the growth of *X*. *citri* pv. *citri* 306. The dark arrow indicates the drop from the highest to the lowest absorbance values after the exponential growth phase. (B) The combined effect of 5 mM CaCl_2_ with varying NaCl concentrations on *X*. *citri* growth. The dashed black square highlights the prevention of the absorbance decline in cultures grown with 5 mM CaCl_2_ jointly with 50, 100, or 250 mM NaCl. Arrows, colored according to the curve scheme, indicate the highest and lowest absorbance values reached under each condition after entering the stationary growth phase. Five experimental replicates were performed.

### Ca^2+^, Mg^2+^, and Mn^2+^ Induce Smaller and More Homogeneous *X*. *citri* Cells

To evaluate the impact of different cations on the cellular morphology, an overnight culture of *X*. *citri* in exponential phase grown in 2xYTON medium at 28 °C under agitation was transferred to new tubes with fresh 2xYTON medium without and with each ion (5 mM), adjusting the OD_600 nm_ to 0.1. After 12 h of incubation in the same conditions, the cells were analyzed by negative stain transmission electron microscopy (TEM). Images of 100 bacteria were captured for each condition (Figure 4).

**Figure 4:**
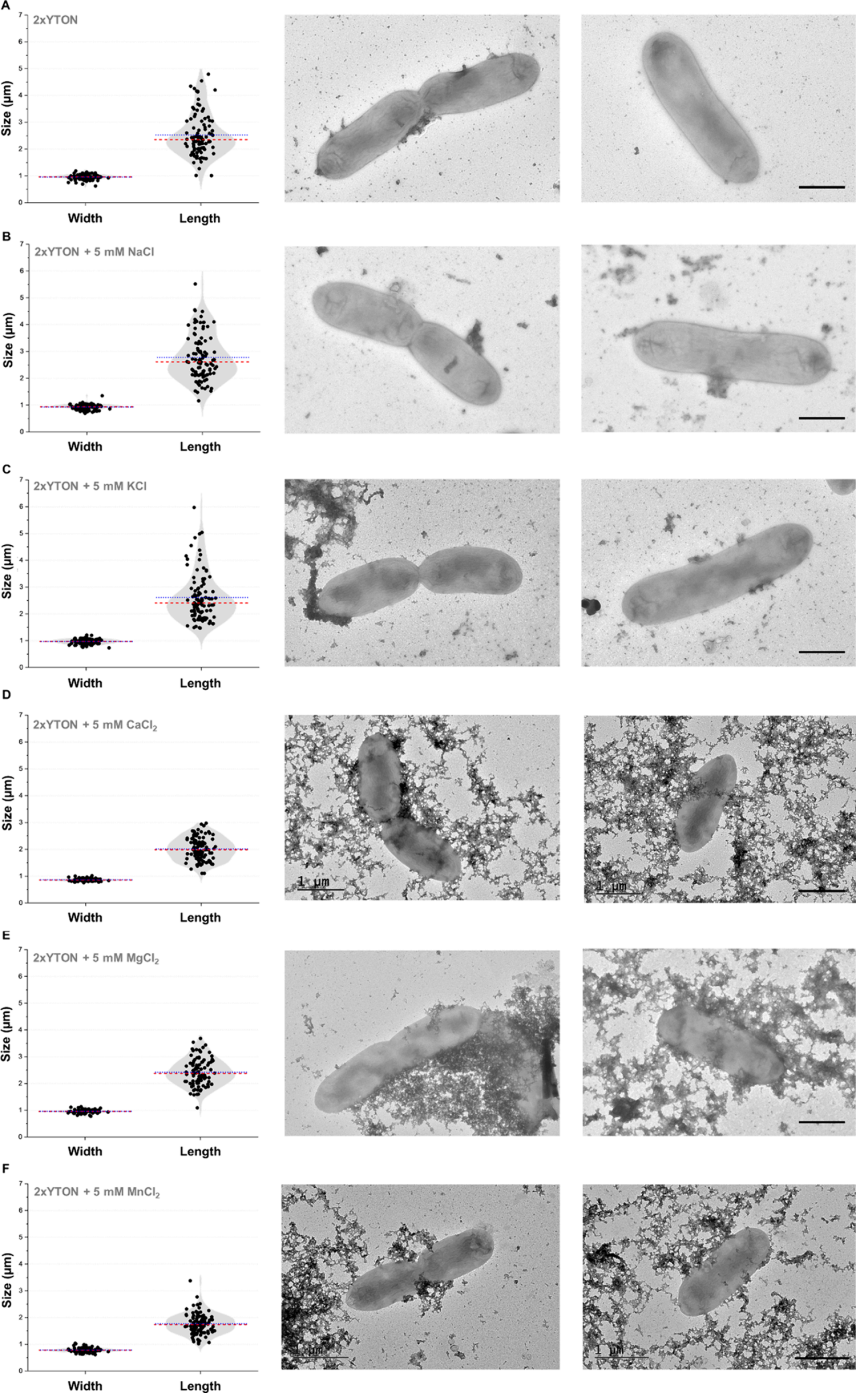
Impact of monovalent and divalent cations on the morphology of *X*. *citri*. *X*. *citri* pv. *citri* 306 cells were incubated for 12 h in 2xYTON medium without ion addition (A) and in 2xYTON medium supplemented with 5 mM NaCl (B), 5 mM KCl (C), 5 mM CaCl_2_ (D), 5 mM MgCl_2_ (E), and 5 mM MnCl_2_ (F). Negative staining was performed using a 2% uranyl acetate solution. 100 cells were used to calculate the parameters shown in panels (A–F). Scale bar = 1 μm.

Cells grown in 2xYTON medium had average dimensions of 960 ± 95 nm in width and 2,526 ± 780 nm in length (Figure 4A). Supplementation with monovalent ions, Na^+^ or K^+^, did not affect bacterial dimensions: 1,011 ± 58 and 965 ± 92 nm in width (Figure 4B) and 2,702 ± 828 and 2,608 ± 878 nm in length (Figure 4C), respectively. Supplementation with divalent cations (Ca^2+^, Mg^2+^, or Mn^2+^), however, resulted in smaller and more homogeneous cells compared to the nonsupplemented medium (Figure 4D). Bacterial dimensions grown in the presence of Ca^2+^, for example, have a 864 ± 45 nm width and a 2,015 ± 425 nm length. Mg^2+^ induced the formation of the largest cells among the three divalent ions tested, with cell sizes ranging from 959 ± 60 nm in width and 2,422 ± 487 nm in length (Figure 4E). In contrast, the addition of Mn^2+^ in 2xYTON medium resulted in the smallest cells, measuring 786 ± 81 nm in width and 1,769 ± 83 nm in length (Figure 4F). The presence of Ca^2+^ and Mn^2+^ resulted in cells approximately 30% shorter in length compared to the control, whereas Mg^2+^ led to only an ∼ 5% reduction in cell length.

### Growth Enhancement by Calcium Is Restricted to the Xanthomonadaceae Family

Since Ca^2+^ supplementation improved *X*. *citri* fitness, we investigated whether this divalent cation-induced growth enhancement could be observed in other bacterial species. For this, Gram-positive and Gram-negative bacteria from different phyla were chosen, including *Staphylococcus aureus* (Firmicutes, Bacilli), *Acinetobacter baumannii*, *Escherichia coli*, *Stenotrophomonas maltophilia* (Pseudomonadota, Gammaproteobacteria), the oligotrophic bacteria *Caulobacter crescentus* (Pseudomonadota, Alphaproteobacteria), and *Leptospira biflexa* (Spirochaetota, Spirochaetia).

The presence of 5 mM Ca^2+^ in the medium had no significant impact on the growth of *S*. *aureus*, except for a slight improvement in growth with 2 M NaCl (Figure 5A). Interestingly, for *Acinetobacter baumannii*, *Escherichia coli*, and *Stenotrophomonas maltophilia*, despite belonging to the same class of Gammaproteobacteria, each of the three bacteria responded differently to the presence of 5 mM Ca^2+^ in the medium. First, for the opportunistic pathogen *A*. *baumannii*, Ca^2+^ had a negative effect, marginally reducing the maximal density reached in culture, being even more affected when the medium was also supplemented with 50 mM NaCl. Nevertheless, calcium was able to slightly improve the growth in 500 mM NaCl (Figure 5B).

**Figure 5:**
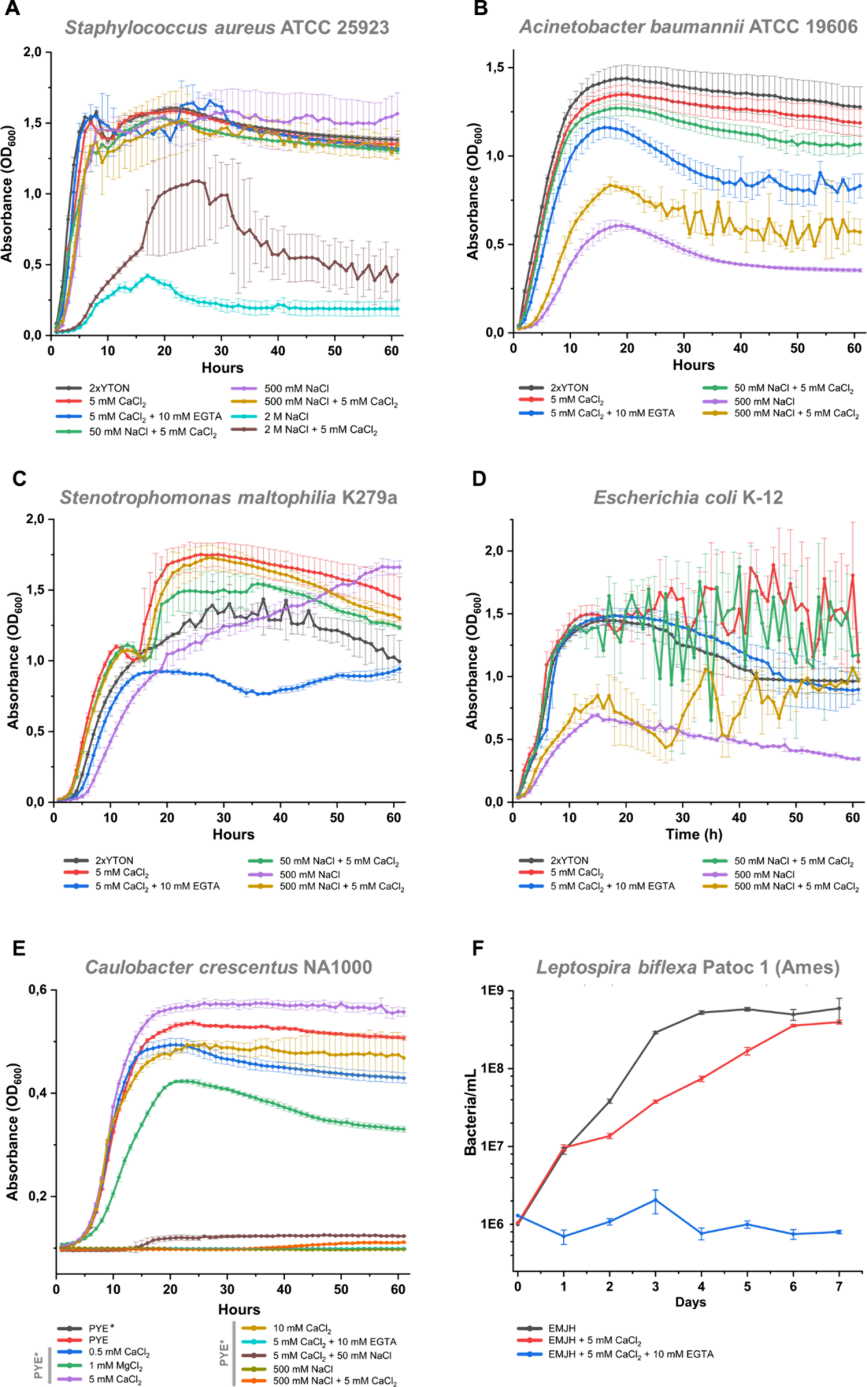
Effects of CaCl_2_, NaCl, and EGTA on the growth of bacterial species from different phyla. Growth curves of *Staphylococcus aureus* ATCC 25923 (A), *Acinetobacter baumannii* ATCC 19606 (B), *Stenotrophomonas maltophilia* K279a (C), and *Escherichia coli* K-12 (D) in 2xYTON medium, *Caulobacter crescentus* NA1000 (E) in PYE (complete) and PYE* (without CaCl_2_ and MgCl_2_) media, and *Leptospira biflexa* sv. *Patoc* (Ames) in EMJH medium supplemented with *Leptospira* Enrichment EMJH (F). A total of three experimental replicates were performed.

*S*. *maltophilia*, an environmental opportunistic pathogen, was tested due to its genetic similarity to *X*. *citri*, both belonging to the Xanthomonadaceae family, revealing a considerable growth enhancement by Ca^2+^ and a complete recovery of growth in 500 mM NaCl supplemented with 5 mM Ca^2+^ (Figure 5C), a phenotype similar to that observed for *X*. *citri*. Interestingly, under the three calcium-supplemented conditions (5 mM CaCl_2_, 5 mM CaCl_2_ + 50 mM NaCl, and 5 mM CaCl_2_ + 500 mM NaCl), the exponential log phase was divided into two parts, separated by a brief lag phase, which was absent in conditions without Ca^2+^ supplementation (Figure 5C). A comparable but more modest growth pattern was observed for *X*. *citri* when cultured in 5 mL of calcium-supplemented medium (Figure 1K). This pattern resembles diauxic growth, which typically exhibits two distinct exponential phases separated by a second lag phase. The diauxic lag occurs as a result of metabolic readaptation, during which the bacteria synthesize the necessary enzymes to utilize a second carbon source once the primary one is depleted.^28^ For *S*. *maltophilia*, the most evident beneficial phenotype was an increase in turbidity, accompanied by a 13% rise in *k*.

For *E*. *coli*, the curves became extremely noisy after reaching the stationary phase due to the drastic change from planktonic to aggregated forms. This suggests that calcium may induce the synthesis of proteins involved in intercellular adhesion (Figure 5D).

Considering that the PYE medium already has CaCl_2_ and MgCl_2_ in the recipe, PYE* was used for *C*. *crescentus* cultivation instead. The cation-lacking medium could not support *C*. *crescentus* growth, while the addition of each ion separately partially recovered the growth, Ca^2+^ more efficiently than Mg^2+^. Increasing the CaCl_2_ concentration from 0.5 to 5 mM could benefit growth, but *C*. *crescentus* was observed to be less tolerant to osmotic pressure once 10 mM CaCl_2_ slightly reduced the maximum density, and any concentration of NaCl resulted in complete or almost complete growth inhibition (Figure 5E). For *L*. *biflexa*, 1 mM calcium retarded growth, while chelation by EGTA inhibited the *in vitro* development; however, some living cells could still be found after 7 days of growth (Figure 5F).

### Calcium Induces Bacterial Adhesion to Abiotic Surfaces

Aggregation phenotypes observed in some bacteria growing in broth medium, e.g., large macroscopic aggregates of *E*. *coli* K-12 in calcium-supplemented 2xYTON (Figure S3) and *X*. *citri* adhered in the air–water interface of shaking Erlenmeyers when incubated with calcium, motivated us to investigate the relation of this cation to surface adhesion and biofilm formation *in vitro*.

All *X*. *citri* strains, WT and mutants (Δ*pilB*, Δ*rpfF*, Δ*gumD*, and Δ*rpfF*Δ*gumD*), failed to adhere to the microplates when incubated in the 2xTY medium. However, when 5 mM CaCl_2_ was added to the growth medium, a clear halo was formed in the air–water interface after crystal violet staining. Therefore, the addition of 5 mM Ca^2+^ resulted in an 8-fold greater bacterial adhesion (from 0.03 to 0.25 in absorbance, Figure 6A). Calcium is known to induce type 4 pilus (T4P) synthesis in *X*. *citri*, which is associated with twitching motility, leaf adhesion, and biofilm formation.^26^ The *X*. *citri* Δ*pilB* mutant, which lacks the T4P polymerization ATPase and therefore cannot extend the filaments, exhibited reduced but not completely abolished adherence to polystyrene compared to *X*. *citri* WT, confirming the importance of the T4P for adhesion but suggesting the presence of other mechanisms mediating surface attachment. Since diffusible signal factor (DSF) is known to lead to biofilm dispersion of *X*. *campestris*,^29^ the QS mutant knockout of the DSF synthase, RpfF, was tested. The Δ*rpfF* mutant exhibited a 2-fold increase in adhesion compared to the *X*. *citri* wild type, rising from 0.25 to 0.5 in absorbance (Figure 6A), suggesting that this phenotype is independent of quorum sensing (QS) but is favored when this system is inactive. Another important component of *Xanthomonas* spp. biofilm is xanthan gum, the major exopolysaccharide produced by this bacterium, which is known to interact with divalent metal.^30,31^ To evaluate whether the adhesion was dependent on xanthan gum production, a Δ*gumD* mutant deficient in xanthan synthesis and the double mutant Δ*rpfF*Δ*gumD* were tested. Both mutants showed adherence levels comparable to those of their parental strains, *X*. *citri* WT and Δ*rpfF*, respectively, suggesting that this *gumD*-dependent exopolysaccharide was not related to this phenotype (Figure 6A).

**Figure 6:**
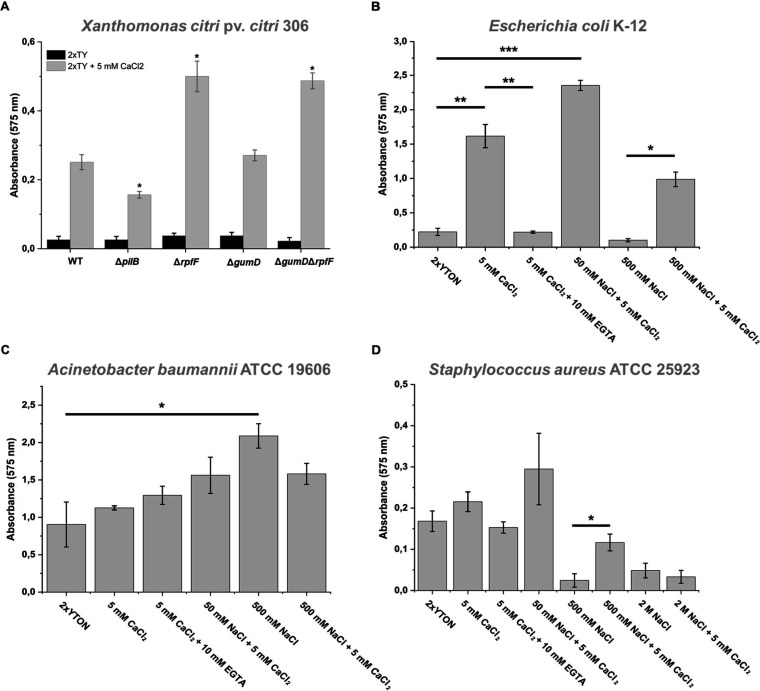
The effect of supplementation of calcium on the growth medium of different bacteria in the polystyrene adhesion microplate. Calcium importance for polystyrene adhesion in the microplate of *Xanthomonas citri* pv. *citri* 306 (A), *Escherichia coli* K-12 (B), *Acinetobacter baumannii* ATCC 19606 (C), and *Staphylococcus aureus* ATCC 25923 (D). *X*. *citri* was incubated at 28 °C for 5 days, while *E*. *coli*, *A*. *baumannii*, and *S*. *aureus* were incubated at 37 °C for 48 h, all in static conditions. *p < 0.05, **p < 0.005, and ***p < 0.0005. A total of three experimental replicates were performed.

Adherence assays were performed for *E*. *coli*, *A*. *baumannii*, and *S*. *aureus*. In the case of *E*. *coli*, the presence of calcium exhibited increased adhesion, particularly when the medium was cosupplemented with 50 mM NaCl. The addition of 10 mM EGTA completely reversed this phenotype (Figure 6B). On the other hand, only a high salt concentration (500 mM NaCl) resulted in increased adhesion of *A*. *baumannii*, which was reverted by cosupplementation with 5 mM CaCl_2_ (Figure 6C). In the case of *S*. *aureus*, the addition of Ca^2+^ did not significantly affect the bacterial adhesion, except when the medium was supplemented with 500 mM NaCl and 5 mM CaCl_2_ (Figure 6D). Once again, *X*. *citri* demonstrated a variety of phenotypes influenced by Ca^2+^ in a manner that is not conserved across different bacterial species.

## Discussion

*X*. *citri* is of great significance to global citriculture and serves as a model organism in phytopathology studies.^32^ Our findings demonstrate that *X*. *citri* exhibited remarkably improved growth in the presence of divalent cations (Ca^2+^, Mg^2+^, Mn^2+^, Fe^2+^, Ni^2+^, and Zn^2+^), whereas monovalent ions (Na^+^, K^+^, and Li^+^) had no noticeable effect. This growth enhancement also correlated with the increased number of viable bacterial cells determined by CFU counting from 5 mL cultures, confirming the faster cell cycle in the presence of all divalent cations tested except Co^2+^. The cellular Ca^2+^ concentration in *E*. *coli* grown in LB medium is maintained at around 100 μM.^33^ However, it is tightly regulated to nanomolar concentrations in the cytoplasm, serving as a messenger through influx and efflux systems, signaling to regulate the protein machinery and gene expression face of environmental stimuli.^15,34,35^ For Mg^2+^, the cytoplasmic storage of this cation can reach 100 mM in *E*. *coli* ^36,37^ and is essential for DNA and ribosome stabilization.^20^ On the other hand, transition metals are found as trace elements. *E*. *coli* cells grown in LB medium were shown to store Fe^2+^, Mn^2+^, Ni^2+^, and Co^2+^ at concentrations of 100, 10, 1, and 0.1 μM, respectively, while Zn^2+^ is also present at 100 μM.^33^ The results presented here reveal the growth of *X*. *citri* can be enhanced by supplementing the medium with most of the divalent metals tested (Ca^2+^, Mg^2+^, Mn^2+^, Fe^2+^, Ni^2+^, and Zn^2+^) at concentrations ranging from micromolar (μM) to millimolar (mM). Such a feature could be related, for instance, to a protein interaction as a cofactor, initiating catalytic or regulatory activities. The addition of calcium to the *X*. *citri* growth medium enhances bacterial fitness by accelerating the cell cycle, likely through increased energy availability or a calcium-driven metabolic reorganization that promotes cell division. However, the underlying mechanisms behind this phenotype remain unclear and warrant further investigation. In bacteria, ATP hydrolysis generates the energy required for direct Ca^2+^ uptake or extrusion through the membrane via specialized transporters, such as Ca^2+^/H^+^ and Ca^2+^/Na^+^ antiporters.^38,39^ Additionally, poly-3-hydroxybutyrate (PHB), synthesized by all living organisms, can assume a complex form with polyphosphate (polyP) that interacts with Ca^2+^, forming the polyP/Ca^2+^/PHB complex.^40^ This nonproteinaceous structure is predominantly localized in mitochondrial membranes and plays a crucial role in Ca^2+^ uptake within this compartment.^41^ This feature is also proposed for bacterial models, where its production is intimately regulated by the presence of Ca^2+^ ions.^42^ In *E*. *coli*, medium supplementation with Ca^2+^ resulted in a differential gene expression, including the upregulation of enzymes involved in the citric acid cycle and with the intermediary metabolism.^43^ Moreover, *Yersinia pestis* ceases growth in a phenomenon known as growth restriction. However, this growth can be restored by the addition of ATP or divalent cations to the medium. This phenotype is dependent on the low-calcium response protein H.^44^

Iron is an essential element for almost all bacteria due to its participation in redox centers of several enzymes from the respiratory chain, photosynthesis, and intermediary metabolism.^45^ However, bacteria must maintain the intracellular fraction of this metal in a nontoxic form to avoid undesired reactions, such as the production of the highly reactive and damaging hydroxyl radical, which can be formed by the interaction of Fe^2+^ with hydrogen peroxide (H_2_O_2_) through the Fenton reaction.^46^ Mn^2+^, another trace element, is essential for most bacterial species, though high concentrations of Mn^2+^ can induce cytotoxic effects as a result of the mismetalation of proteins.^47^ Nevertheless, for species that evolved a manganese-centric metabolism, such as *Borrelia* sp. and *Lactobacillus* sp. where little or no Fe^2+^ is needed for growth, increased concentrations of Mn^2+^ are naturally found.^23,24^ Nickel functions as a cofactor of metalloenzymes that participates in energy and nitrogen metabolism, such as ureases, Fe–Ni hydrogenases, carbon monoxide dehydrogenase, superoxide dismutase SodN, methyl-coenzyme M reductase, glyoxalase I, and acetyl-coenzyme A decarbonylase/synthase.^48–52^ In addition to metalloenzymes, these cations can also be coordinated as metallic centers in structurally complex cofactors, such as chlorophyll (Mg^2+^), heme (Fe^2+^), cofactor F430 (Ni^2+^), or cobalamin (Co^3+^).^53^

*Xanthomonas* spp. use these cations for several cellular processes, as well. Disturbances in ion homeostasis controlling systems were shown to greatly impact bacterial development *in vitro* and *in vivo*. In *X*. *oryzae*, Mn^2+^ influx and efflux are mediated by MntH and YebN proteins, respectively. The mutant for the *yebN* gene was shown to be less virulent and more sensitive to high concentrations of Mn^2+^ in the medium.^54^ Iron and nickel uptake systems FeoB and *zur* (zinc uptake regulator), respectively, were also essential for plant virulence in *X*. *oryzae* and *X*. *campestris*.^55–57^ Additionally, *Xanthomonas* spp. count with the PhoPQ two-component system to modulate gene expression in Ca^2+^ and Mg^2+^ limiting conditions. These genes are responsible for the expression of HrpG, AvrXA21 activity, and virulence in *X*. *oryzae* ^58^ and were found to be essential for *X*. *campestris* growth.^59^ Additionally, divalent metals can also bind to xanthan gum disaccharide units in a 1:2 stoichiometry.^31^ Such an interaction is important to assist in host colonization. *X*. *campestris* invades leaf epidermis through open stomata or accidental wounds.^60^ While increases in K^+^ concentration stimulate stomatal opening, elevated cytosolic Ca^2+^ triggers stomatal closure.^61^ Therefore, the ability of xanthan to chelate divalent cations enhances bacterial virulence by disturbing innate immunity through the suppression of MAMP (microbial associated molecular pattern)-induced signaling^62^ and modulating stomatal closure,^63^ both mechanisms mediated by Ca^2+^ influx. Despite its importance for *in vivo* development, the *in vitro* growth improvement of *X*. *citri* by Ca^2+^ supplementation was shown to be independent of xanthan since the Δ*gumD* mutant could also benefit from Ca^2+^ ions for growth enhancement. While divalent cations can stimulate bacterial virulence, previous studies have shown that the expression of some virulence factors can be suppressed in high concentrations of NaCl, such as the *hrp* (hypersensitive reaction and pathogenicity) gene.^64^

Despite their biological importance, transition metals have also been studied for application as controlling agents against phytopathogens. The Bordeaux mixture was the first copper-based antimicrobial compound created and used to combat the downy mildew disease caused by *Plasmopara viticola*.^65^ New formulations were designed, such as copper oxychloride, copper hydroxide, and cuprous oxide, and are currently the most used alternative to deal with citrus canker disease worldwide.^66^ However, several reports discuss the emergence of copper-resistant (Cu^R^) *Xanthomonas*, in which copper sprays are inefficient. This resistance is usually related to plasmidial genes that allow cellular detoxification through ion sequestration or elimination through efflux systems.^66–68^ Diverse successful strategies have been developed to fight against Cu^R^ *Xanthomonas* species, for instance, hybrid magnesium–copper nanomaterials,^69^ zinc thiazole compounds,^70^ bifunctional peptides,^71^ piperidine and pyrrolidine,^72^ and bacteriophage cocktails.^73,74^ The use of dissolved zinc thiazole in DMSO at 76 μM significantly inhibited the growth of *X*. *oryzae* pv. *oryzae*. Interestingly, zinc thiazole at 76 μM seems to enhance the cell division and alter the cell wall integrity of the bacteria.^70^

It was surprising that *X*. *citri* pv. *citri* strain 306 not only tolerated but also benefited from relatively high concentrations of other cations known to induce oxidative stress, such as Zn^2+^, Ni^2+^, and Fe^2+^, which exhibited toxicity only at concentrations above 1 mM. Apart from their enzymatic importance, divalent cations can also participate in the stabilization of bacterial membranes and cell walls.^75,76^ This interaction is primarily described for Ca^2+^ and Mg^2+^ cations, although Mn^2+^ was also shown to interact with peptidoglycan *in vitro*.^77^ In Gram-negative bacteria, the lipopolysaccharide is also stabilized by the presence of metals such as Ca^2+^ and Mg^2+^. When available in the extracellular milieu, divalent cations can almost completely replace monovalent ions, creating much more robust structures. The absence of divalent cations negatively affected the outer membrane and resulted in a disordered bilayer with phospholipids and LPS mixed in both the inner and outer leaflets.^5,78^ Coincidently, calcium chelation resulted in morphological changes from the L-form to spherical shaped *E*. *coli*, vacuole formation, and lysis.^79^ Additionally, some authors have also described a protective effect of divalent cations against antimicrobial agents, such as polymyxin B, polyphenols, and cationic peptides.^80–82^ For *X*. *citri*, negative staining TEM image analysis revealed a homogeneous morphology of smaller cells grown in 2xYTON supplemented with any of the divalent metals, whereas cells grown with monovalent cations, as well as 2xYTON without ion supplementation, were larger and heterogeneous in size. Stabilization of cellular structures by interaction with divalent cations, such as the LPS, might explain the morphological changes in each condition, although this role is especially played by Ca^2+^ and Mg^2+^, which is not yet described for Mn^2+^. It is important to note that smaller cells are more efficient at absorbing nutrients from the extracellular environment due to their higher surface-to-volume ratio. New assays are necessary to gain deeper insight into the biological effects of divalent cations on the growth of *Xanthomonas* species.

Growth enhancement was also observed for *X*. *campestris* and *S*. *maltophilia* incubated in the medium with the presence of 5 mM CaCl_2_, both belonging to the Xanthomonadaceae family. Wondering whether other bacteria phylogenetically distant from *Xanthomonas* species could also benefit from divalent metals for growth improvement, we tested members of a broad range of families. However, none of them exhibited significantly better growth in the presence of calcium compared to the Ca^2+^-free medium, while this cation had, actually, a negative influence on the growth of *L*. *biflexa*. Still, Ca^2+^ was at least slightly important for growth in high salt concentration for almost all bacteria tested in this work.

Bacteria can adapt to environmental changes using protein systems that respond to fluctuations in ion concentration. Here, we observed an aggregative phenotype of *X*. *citri* and *E*. *coli* grown in the presence of calcium, forming a clear line of adherent cells in the air–water interface. Biofilm assays were performed to explore the sessile behavior. The addition of 5 mM Ca^2+^ resulted in an 8-fold increase in bacterial adhesion. In the case of the *X*. *citri* Δ*pilB* mutant, which lacks the ATPase required for T4P polymerization and is therefore unable to produce this extracellular filament involved in adhesion and twitching motility, it exhibited reduced adherence when grown in a medium supplemented with Ca^2+^ ^26,83^ compared to *X*. *citri* WT. However, adherence was not completely abolished, suggesting the involvement of other calcium-dependent adhesins in the process. In contrast, the adherence of the Δ*rpfF* mutant unable to activate the QS was two times higher in comparison to that of WT.

QS is known to control the expression of a broad range of genes, including adhesins.^84^ Chatterjee and collaborators^85^ reported that gene coding for FimA, HxfA, and HxfB adhesins were upregulated in the *Xylella fastidiosa* Δ*rpfC* mutant. Curiously, Li and collaborators observed a reduction in biofilm formation *in vitro* (polypropylene surface) and *in vivo* (Duncan grapefruit leaves) by the Δ*rpfF* mutant of *X*. *citri* cultured in the XVM2 medium that also contained calcium in its composition.^86^ However, Crossman et al. reported that Δ*rpfF* mutants of *X*. *campestris* also exhibited increased adhesion to plastic in the first hours of incubation compared to WT independently of xanthan gum production, similar to our results.^87^ Xanthan gum is the main exopolysaccharide produced by *Xanthomonas* spp. and is the major constituent of its biofilm mass. It is known that the high amount of xanthan gum produced during bacterial growth is dependent on QS, although Δ*rpfF* mutants can also synthesize small amounts of this exopolysaccharide that are essential for intercellular adhesion and formation of the typical aggregates observed in these mutants.^29^ As expected, our *X*. *citri* double mutant Δ*rpfF*Δ*gumD* lost the ability to grow in aggregate forms. In agreement with the literature, the Δ*gumD* and Δ*rpfF*Δ*gumD* mutants exhibited adhesion profiles similar to those of the parental strains *X*. *citri* WT and Δ*rpfF*, respectively, indicating that xanthan gum was not essential for *in vitro* adherence under the tested conditions.

For the other bacteria tested, only *E*. *coli* K-12 adherence was strictly dependent on Ca^2+^, whereas *S*. *aureus* was favored by Ca^2+^ only when coincubated with 500 mM NaCl. The adherence behavior of *A*. *baumannii* was completely independent of calcium. There are many reports in the literature describing different mechanisms of calcium participating in adhesion and biofilm formation for a wide range of bacteria to biotic and abiotic surfaces.

Ca^2+^, but not Mg^2+^ and Mn^2+^, significantly increased the binding of *Lactobacillus* spp. to jejunal epithelial cells IPEC-J2, avoiding the adhesion of *E*. *coli* to this tissue.^88^ Similarly, adhesion of Enterococci to IPEC-J2 cells increased to around 55% in the presence of calcium,^89^ and calcium chelation reduced the adhesion of *Lactobacillus* spp. to human enterocyte-like Caco-2 cells.^90^ *Shigella flexneri* adhesion to guinea pig intestinal cells was also dependent on Ca^2+^, which could not be replaced for Mg^2+^ or Mn^2+^.^91^ For *Pseudomonas aeruginosa*, biofilm rate and extent were reported to increase proportionally with the calcium concentration.^92^ In addition to the biological material, such as the extracellular matrix mainly composed of exopolysaccharide, regulated mineralization was shown to be essential for biofilm structuring by calcium carbonate production in *P*. *aeruginosa*, *Bacillus subtilis*, and *Mycobacterium smegmatis* cultures,^93,94^ highlighting the importance of this cation not only for direct bacterial adhesion to surfaces but also for the maintenance of microbiological communities in nature.

## Conclusion

In this study, we show that the supplementation of growth media with divalent cations significantly enhances *X*. *citri* growth fitness by shortening the lag phase, reducing generation time, increasing cell density, increasing bacterial homogeneity, and decreasing the bacterial size. Among the cations tested, Ca^2+^ and Fe^2+^ showed the most pronounced effects, with CaCl_2_ increasing the specific growth rate by up to 223.5%. The molecular basis of this phenotype remains to be elucidated and requires further investigation. Based on these results, the study proposes an optimized medium, called 2xTY-Ca, consisting of 2xTY medium (16 g/L tryptone, 10 g/L yeast extract, 5 g/L NaCl) with 1 mM CaCl_2_, as a simple and effective formulation to promote the robust growth of *X*. *citri* and *X*. *campestris* under laboratory conditions. Future research should aim to elucidate the metabolic adaptations of these microorganisms to divalent cations and explore their biotechnological potential, particularly in fields such as xanthan gum production. *X*. *campestris* is of industrial importance due to its ability to produce xanthan gum, a polysaccharide widely used as a stabilizing and thickening agent. This dual role underscores the need to optimize growth conditions for *Xanthomonas* species, both as a model organism for structural and biological studies and as a significant phytopathogen. Such optimization could lead to major advancements in scientific understanding and industrial applications.

## Supplementary Material



## Notes

Additional growth curves for *X*. *citri* and *X*. *campestris*, images of *E*. *coli* cultivation, and lag phase and generation times for various bacteria (PDF)
