# Supplementary material for: Divalent Cations (Ca2+, Mg2+, Mn2+, Fe2+, Ni2+, and Zn2+) Enhance Growth of Xanthomonas citri and X. campestris by Reducing Generation Time
Source: ACS Omega. 2025 Aug 6;10(32):35827–41. doi: 10.1021/acsomega.5c02786 (PMC12368633; doi:10.1021/acsomega.5c02786)
Supplement: Supplementary file 1 [file ao5c02786_si_001.pdf]

Divalent Cations (Ca^{2+}, Mg^{2+}, Mn^{2+}, Fe^{2+}, Ni^{2+}, and Zn^{2+}) Enhance Growth of *Xanthomonas citri* and *X. campestris* by Reducing Generation Time

Davi Gabriel Salustiano Merighi[1], Cauê Augusto Boneto Gonçalves[1], Anielle Salviano de Almeida Ferrari[1], Maxuel de Oliveira Andrade[2], Cristiane Rodrigues Guzzo[1*]

[1] Department of Microbiology, Institute of Biomedical Sciences, University of São Paulo, São Paulo CEP 05508-000, Brazil.
[2] Brazilian Biorenewables National Laboratory (LNBR), Brazilian Center for Research in Energy and Materials (CNPEM), Campinas, Brazil.

*Corresponding author
E-mail: crisguzzo@usp.br and crisguzzo@gmail.com

To whom correspondence should be addressed:

Cristiane R. Guzzo, Ph.D, Department of Microbiology, Institute of Biomedical Sciences, University of São Paulo, Av. Prof. Lineu Prestes, 1374, Cidade Universitária, 05508-000, São Paulo/SP, Brazil, +55 11 3091-7298; E-mail: crisguzzo@usp.br

Index

Figure S1. Growth curves of *X. citri* in the presence and absence of cation.. .**Error! Bookmark not defined.**

Figure S2. Effect of Ca^{2+} in the growth of *Xanthomonas campestris* pv. campestris ATCC 33913.. 2

Figure S3. *E. coli K-12* cultivated in 96 wells microplates without and with $CaCl_2$...................3

Table S1. Lag phase duration and generation time of *X. citri* in 2xTY. 3

Table S2. Lag phase duration and generation time of *X. citri* in 2xYTON. 4

Table S3. Lag phase duration and generation time of *X. citri* in 2xYTON with NaCl. 4

Table S4. Lag phase duration and generation time of *X. citri* in 2xYTON with LiCl. 5

Table S5. Lag phase duration and generation time of *X. citri* in 2xYTON with $CaCl_2$. **5**

Table S6. Lag phase duration and generation time of *X. citri* in 2xYTON $MgCl_2$. 6

Table S7. Lag phase duration and generation time of *X. citri* in 2xYTON with $MnCl_2$. 6

Table S8. Lag phase duration and generation time of *X. citri* in 2xYTON with $FeCl_2$..............7

Table S9. Lag phase duration and generation time of *X. citri* in 2xYTON with $NiCl_2$. 7

Table S10. Lag phase duration and generation time of *X. citri* in 2xYTON with $ZnCl_2$............8

Table S11. Lag phase duration and generation time of *X. citri* in 2xYTON with $CoCl_2$. 8

Table S12. Lag phase duration and generation time of *X. citri* in 2xTY and 2xYTON with and without $CaCl_2$. 9

Table S13. Lag phase duration and generation time of *S. aureus*. 9

Table S14. Lag phase duration and generation time of *A. baumannii*. 10

Table S15. Lag phase duration and generation time of *S. maltophilia*. 10

Table S16. Lag phase duration and generation time of *E. coli*...11

Table S17. Lag phase duration and generation time of *C. crescentus*. 11

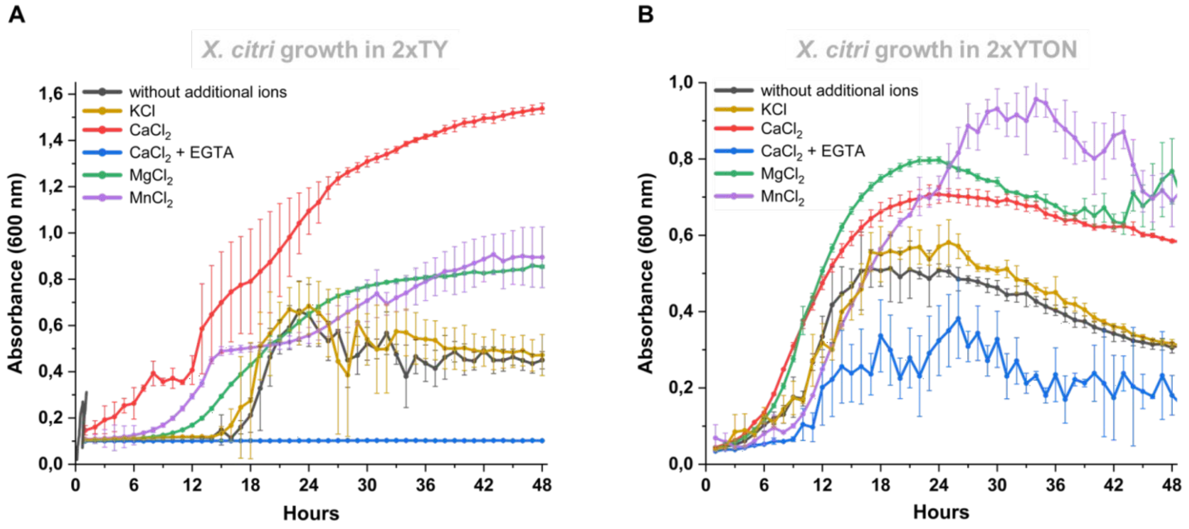

Figure S1. Growth curves of *X. citri* in the presence and absence of cation. *X. citri* pv. *citri* 306 growth curves in 2xTY (**A**) and 2xYTON (**B**) media supplemented with 5 mM monovalent (Na^+ and K^+) or divalent (Ca^{2+}, Mg^{2+}, and Mn^{2+}) cations. A total of three experimental replicates were performed.

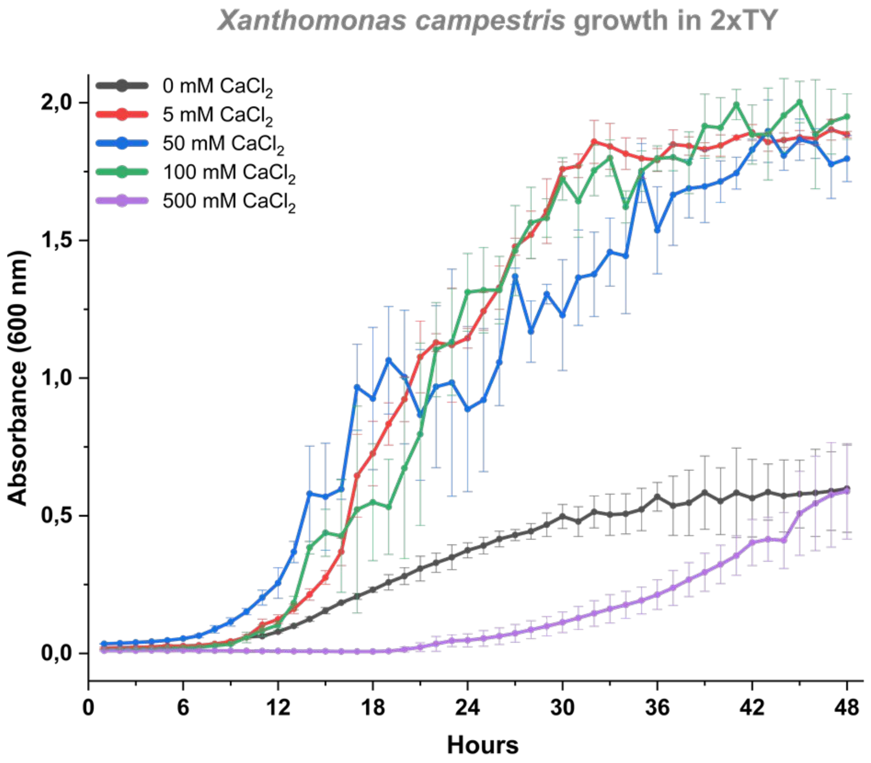

Figure S2. Effect of Ca^{2+} in the growth of *Xanthomonas campestris* pv. *campestris* ATCC 33913. *X. campestris* was grown in 2xTY prepared in 96-well microplate, incubated under orbital agitation 282 cpm at 28 °C for 48h. A total of three experimental replicates were performed.

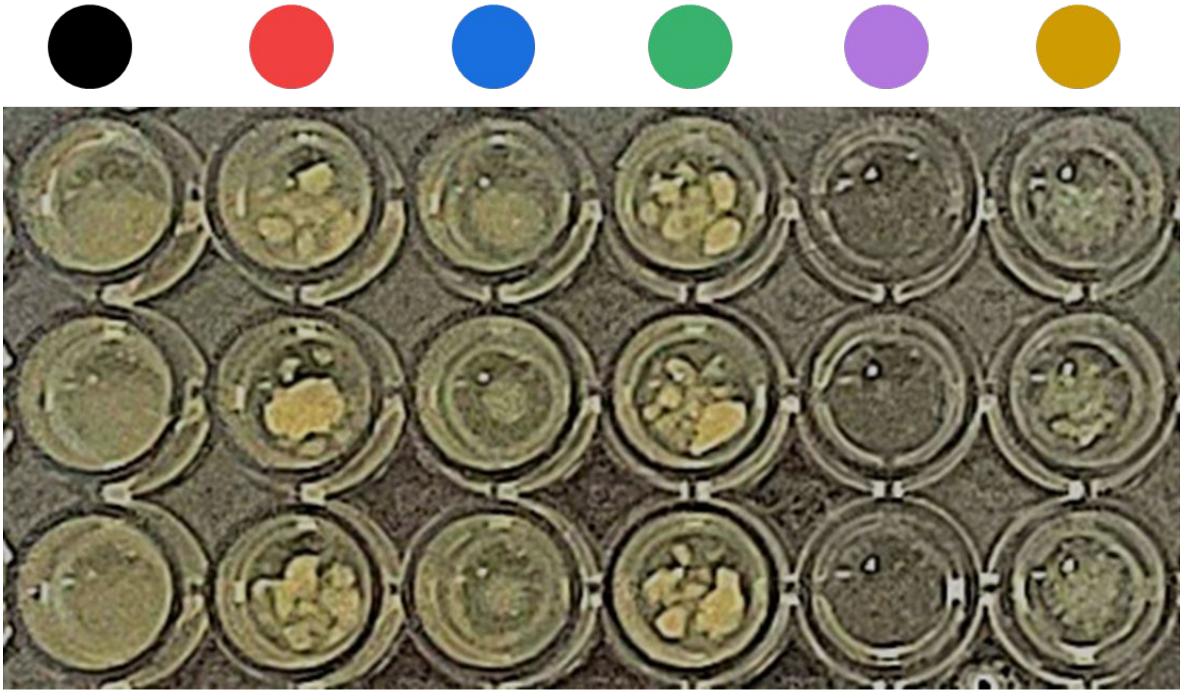

Figure S3. ***E. coli*** **K-12 cultivated in 96 wells microplates without and with $CaCl_2$.** Aggregative phenotype exhibited by *Escherichia coli* K-12 when incubated in the presence of Ca^{2+} during the growth curve experiment plotted in **Figure 3D** from the main article.

Table S1. Lag phase duration and generation time of *X. citri* in 2xTY.
Lag time and generation time (*g*) of *X. citri* pv. *citri* 306 calculated from **Figure S1-A**.

	Lag phase	Generation time (g)
**2xTY**	15 h	2,2 ± 1,2 h
**2xTY + 5 mM KCl_2**	15 h	2,4 ± 1,0 h
**2xTY + 5 mM $CaCl_2$**	0 h	8,6 ± 1,1 h
**2xTY + 5 mM $CaCl_2$ + 10 mM EGTA**	no growth	no growth
**2xTY + 5 mM $MgCl_2$**	10 h	5,6 ± 0,2 h
**2xTY + 5 mM $MnCl_2$**	6 h	4,3 ± 0,1 h

Table S2. Lag phase duration and generation time of *X. citri* in 2xYTON.
Lag time and generation time (*g*) of *X. citri* pv. *citri* 306 calculated from **Figure S1-B**.

	Lag phase	Generation time (g)
**2xYTON**	0 h	3,0 ± 0,4 h
**2xYTON + KCl**	0 h	3,1 ± 0,3 h
**2xYTON + $CaCl_2$**	0 h	2,5 ± 0,0 h
**2xYTON + $CaCl_2$ + EGTA**	9 h	6,1 ± 0,5 h
**2xYTON + $MgCl_2$**	0 h	2,7 ± 0,1 h
**2xYTON + $MnCl_2$**	8 h	2,7 ± 0,1 h

Table S3. Lag phase duration and generation time of *X. citri* in 2xYTON with NaCl.
Lag time and generation time (*g*) of *X. citri* pv. *citri* 306 grown with NaCl calculated from **Figure 1A**.

Sodium	Lag phase	Generation time (g)
**0 µM (control)**	0 h	4,3 ± 0,1 h
**100 nM**	0 h	5,1 ± 0,7 h
**1 µM**	0 h	4,4 ± 0,2 h
**10 µM**	0 h	4,8 ± 0,2 h
**100 µM**	0 h	4,7 ± 0,2 h
**1 mM**	0 h	4,5 ± 0,0 h
**10 mM**	0 h	4,4 ± 0,0 h

Table S4. Lag phase duration and generation time of *X. citri* in 2xYTON with LiCl. Lag time and generation time (*g*) of *X. citri* pv. *citri* 306 grown with LiCl calculated from **Figure 1C**.

Lithium	Lag phase	Generation time (g)
**0 µM (control)**	0 h	4,2 ± 0,1 h
**100 nM**	0 h	3,7 ± 0,4 h
**1 µM**	0 h	4,1 ± 0,3 h
**10 µM**	0 h	3,9 ± 0,0 h
**100 µM**	0 h	3,9 ± 0,4 h
**1 mM**	0 h	4,2 ± 0,0 h
**10 mM**	0 h	4,2 ± 0,0 h

Table S5. Lag phase duration and generation time of *X. citri* in 2xYTON with $CaCl_2$. Lag time and generation time (*g*) of *X. citri* pv. *citri* 306 grown with $CaCl_2$ calculated from **Figure 1D**.

Calcium	Lag phase	Generation time (g)
**0 µM (control)**	5 h	5,6 ± 0,4 h
**50 µM**	5 h	5,2 ± 0,6 h
**100 µM**	5 h	4,7 ± 0,3 h
**250 µM**	5 h	3,7 ± 0,0 h
**500 µM**	5 h	3,3 ± 0,0 h
**750 µM**	5 h	3,3 ± 0,0 h
**1.000 µM**	5 h	3,3 ± 0,0 h

Table S6. Lag phase duration and generation time of *X. citri* in 2xYTON $MgCl_2$.
Lag time and generation time (*g*) of *X. citri* pv. *citri* 306 grown with $MgCl_2$ calculated from **Figure 1E**.

Magnesium	Lag phase	Generation time (g)
**0 µM (control)**	4 h	4,6 ± 0,1 h
**50 µM**	0 h	3,8 ± 0,3 h
**100 µM**	0 h	4,7 ± 0,7 h
**250 µM**	0 h	3,5 ± 0,0 h
**500 µM**	0 h	3,4 ± 0,1 h
**750 µM**	0 h	3,6 ± 0,0 h
**1.000 µM**	4 h	3,9 ± 0,0 h

Table S7. Lag phase duration and generation time of *X. citri* in 2xYTON with $MnCl_2$.
Lag time and generation time (*g*) of *X. citri* pv. *citri* 306 grown with $MnCl_2$ calculated from **Figure 1F**.

Manganese	Lag phase	Generation time (g)
**0 µM (control)**	7 h	4,4 ± 0,1 h
**50 µM**	6 h	2,3 ± 0,7 h
**100 µM**	2 h	2,6 ± 0,8 h
**250 µM**	2 h	3,2 ± 0,1 h
**500 µM**	6 h	3,6 ± 0,1 h
**750 µM**	6 h	4,4 ± 0,1 h
**1.000 µM**	6 h	4,5 ± 0,3 h

Table S8. Lag phase duration and generation time of *X. citri* in 2xYTON with $FeCl_2$. Lag time and generation time (*g*) of *X. citri* pv. *citri* 306 grown with $FeCl_2$ calculated from **Figure 1G**.

Iron	Lag phase	Generation time (g)
**0 µM (control)**	4 h	6,3 ± 2,0 h
**50 µM**	4 h	6,1 ± 0,2 h
**100 µM**	4 h	5,4 ± 0,0 h
**250 µM**	4 h	4,2 ± 0,1 h
**500 µM**	4 h	3,7 ± 0,0 h
**750 µM**	5 h	3,2 ± 0,1 h
**1.000 µM**	5 h	3,4 ± 0,0 h

Table S9. Lag phase duration and generation time of *X. citri* in 2xYTON with $NiCl_2$. Lag time and generation time (*g*) of *X. citri* pv. *citri* 306 grown with $NiCl_2$ calculated from **Figure 1H**.

Nickel	Lag phase	Generation time (g)
**0 µM (control)**	0 h	5,6 ± 0,6 h
**50 µM**	0 h	6,5 ± 0,1 h
**100 µM**	0 h	3,0 ± 0,2 h
**250 µM**	0 h	3,9 ± 0,1 h
**500 µM**	0 h	3,3 ± 0,0 h
**750 µM**	0 h	3,2 ± 0,1 h
**1.000 µM**	0 h	3,3 ± 0,0 h

Table S10. Lag phase duration and generation time of *X. citri* in 2xYTON with $ZnCl_2$. Lag time and generation time (*g*) of *X. citri* pv. *citri* 306 grown with $ZnCl_2$ calculated from **Figure 1I**.

Zinc	Lag phase	Generation time (g)
**0 µM (control)**	7 h	4,2 ± 0,1 h
**50 µM**	7 h	4,6 ± 0,0 h
**100 µM**	5 h	6,2 ± 0,4 h
**250 µM**	5 h	6,6 ± 0,2 h
**500 µM**	5 h	3,6 ± 0,1 h
**750 µM**	5 h	3,2 ± 0,0 h
**1.000 µM**	5 h	3,2 ± 0,0 h

Table S11. Lag phase duration and generation time of *X. citri* in 2xYTON with $CoCl_2$. Lag time and generation time (*g*) of *X. citri* pv. *citri* 306 grown with $CoCl_2$ calculated from **Figure 1J**.

Cobalt	Lag phase	Generation time (g)
**0 µM (control)**	7 h	5,7 ± 0,9 h
**50 µM**	7 h	3,9 ± 0,5 h
**100 µM**	5 h	3,3 ± 0,2 h
**250 µM**	5 h	4,0 ± 0,8 h
**500 µM**	4 h	3,5 ± 0,2 h
**750 µM**	4 h	3,3 ± 0,1 h
**1.000 µM**	4 h	3,6 ± 0,0 h

Table S12. Lag phase duration and generation time of *X. citri* in 2xTY and 2xYTON with and without $CaCl_2$.
Lag time and generation time (*g*) of *X. citri* pv. *citri* 306 calculated from **Figure 1K** from main article.

	Lag phase	Generation time (g)
**2xTY**	10 h	5,5 ± 0,8 h
**2xTY + $CaCl_2$**	7.5 h	1,8 ± 0,2 h
**2xYTON**	0 h	3,2 ± 0,5 h
**2xYTON + $CaCl_2$**	0 h	1,7 ± 0,2 h

Table S13. Lag phase duration and generation time of *S. aureus*.
Lag time and generation time (*g*) *Staphylococcus aureus* ATCC 25923 calculated from **Figure 5A** from main article.

	Lag phase	Generation time (g)
**2xYTON**	0 h	1,7 ± 0,1 h
**5 mM $CaCl_2$**	0 h	1,7 ± 0,1 h
**5 mM $CaCl_2$ + 10 mM EGTA**	0 h	1,9 ± 0,1 h
**50 mM NaCl + 5 mM $CaCl_2$**	0 h	1,9 ± 0,1 h
**500 mM NaCl**	0 h	1,2 ± 0,1 h
**500 mM NaCl + 5 mM $CaCl_2$**	0 h	1,2 ± 0,1 h
**2 M NaCl**	3 h	2,9 ± 0,3 h
**2 M NaCl + 5 mM $CaCl_2$**	3 h	2,7 ± 0,1 h

Table S14. Lag phase duration and generation time of *A. baumannii*.
Lag time and generation time (*g*) *Acinetobacter baumannii* ATCC 19606 calculated from **Figure 5B** from main article.

	Lag phase	Generation time (g)
**2xYTON**	0 h	1,8 ± 0,1 h
**5 mM $CaCl_2$**	0 h	1,9 ± 0,1 h
**5 mM $CaCl_2$ + 10 mM EGTA**	0 h	1,8 ± 0,1 h
**50 mM NaCl + 5 mM $CaCl_2$**	0 h	1,7 ± 0,1 h
**500 mM NaCl**	3 h	3,0 ± 0,4 h
**500 mM NaCl + 5 mM $CaCl_2$**	2 h	2,6 ± 0,3 h

Table S15. Lag phase duration and generation time of *S. maltophilia*.
Lag time and generation time (*g*) *Stenotrophomonas maltophilia* K279a calculated from **Figure 5C** from main article.

	Lag phase	Generation time (g)
**2xYTON**	3 h	1,7 ± 0,1 h
**5 mM $CaCl_2$**	0 h	1,5 ± 0,1 h
**5 mM $CaCl_2$ + 10 mM EGTA**	4 h	4,6 ± 0,6 h
**50 mM NaCl + 5 mM $CaCl_2$**	0 h	1,5 ± 0,0 h
**500 mM NaCl**	5 h	2,6 ± 0,1 h
**500 mM NaCl + 5 mM $CaCl_2$**	1 h	1,6 ± 0,0 h

Table S16. Lag phase duration and generation time of *E. coli*.
Lag time and generation time (*g*) *Escherichia coli K-12* calculated from **Figure 5D** from main article.

	Lag phase	Generation time (g)
**2xYTON**	0 h	2,1 ± 0,2 h
**5 mM $CaCl_2$**	0 h	2,2 ± 0,2 h
**5 mM $CaCl_2$ + 10 mM EGTA**	0 h	2,6 ± 0,2 h
**50 mM NaCl + 5 mM $CaCl_2$**	0 h	2,5 ± 0,3 h
**500 mM NaCl**	3 h	2,7 ± 0,1 h
**500 mM NaCl + 5 mM $CaCl_2$**	2 h	2,5 ± 0,1 h

Table S17. Lag phase duration and generation time of *C. crescentus*.
Lag time and generation time (*g*) *Caulobacter crescentus* NA100 calculated from **Figure 5E** from the main article.

	Lag phase	Generation time (g)
**PYE***	no growth	no growth
**PYE**	3 h	3,1 ± 0,1 h
**0.5 mM $CaCl_2$**	3 h	3,0 ± 0,1 h
**1 mM $MgCl_2$**	4 h	4,9 ± 0,1 h
**5 mM $CaCl_2$**	3 h	3,0 ± 0,0 h
**10 mM $CaCl_2$**	3 h	2,7 ± 0,0 h
**5 mM $CaCl_2$ + 10 mM EGTA**	no growth	no growth
**5 mM $CaCl_2$ + 50 mM NaCl**	almost no growth	almost no growth
**0 mM $CaCl_2$ + 500 mM NaCl**	no growth	no growth
**5 mM $CaCl_2$ + 500 mM NaCl**	no growth	no growth
